# Evaluation of antibiotic resistance dissemination by wastewater treatment plant effluents with different catchment areas in Germany

**DOI:** 10.1038/s41598-020-65635-4

**Published:** 2020-06-02

**Authors:** Johannes Alexander, Norman Hembach, Thomas Schwartz

**Affiliations:** Karlsruhe Institute of Technology (KIT), Institute of Functional Interfaces, Hermann-von-Helmholtz Platz 1, D-76344 Eggenstein-Leopoldshafen, Germany

**Keywords:** Microbiology, Molecular biology, Environmental sciences, Natural hazards

## Abstract

The study quantified the abundances of antibiotic resistance genes (ARGs) and facultative pathogenic bacteria (FPB) as well as one mobile genetic element in genomic DNA via qPCR from 23 different wastewater treatment plant (WWTP) effluents in Germany. 12 clinically relevant ARGs were categorized into frequently, intermediately, and rarely occurring genetic parameters of communal wastewaters. Taxonomic PCR quantifications of five FPB targeting *Escherichia coli*, *Pseudomonas aeruginosa*, *Klebsiella pneumoniae*, *Acinetobacter baumannii*, and enterococci were performed. The WWTPs differed in their catchment areas being impacted by hospitals, food processing companies, or housing areas only. The total discharges of the analyzed ARGs and FPB were found to cluster independently of the sizes of the WWTPs with a maximum difference of two log units within one cluster. Initially, quantitative data evaluations revealed no significant difference between ARG categories and WWTP catchment areas. More distinct correlations became obvious with a Pearson correlation approach, where each single taxonomic marker is compared to each ARG target. Here, increased correlation of FPB (i.e. *E. coli*, *K. pneumoniae*, *P. aeruginosa*, and enterococci) with clinically relevant ARGs of the category of rarely occurring resistance genes (*bla*_NDM-1_, *van*A) was found in WWTP effluents being influenced by hospital wastewaters.

## Introduction

The increase in infections caused by antibiotic resistant bacteria (ARB) is depicted to be one of the most important issues for the healthcare system of the 21^th^ century^1^. In the timeframe of 2014 and 2016, approximately one million people died due to infections caused by antibiotic resistant bacteria through inefficient treatment, or were untreatable from the start. This situation is becoming more serious considering the current and future antibiotic drug development. Continuing this situation, projection models indicate a significant increase in ARB-related deaths over the next decades^2^. Although the evolution of antibiotic resistance and ARG-associated gene transfer events is complex, the excessive use and often misuse of antibiotics (AB) in human and veterinary medicine are assumed to be the main driving force for resistance development^3^. In addition, the mobilization of ARGs makes it very difficult to efficiently eliminate ARB once the transmission starts to spread.

This process is enhanced by high bacteria cell densities in nutrient-rich environments^4^. The activated sludge system of WWTPs is by default the perfect setting for ARG transfer events^5–8^. Because WWTPs are not designed to eliminate ARGs and ARBs, the finale discharge into the receiving water is a key mechanism by which ARGs and ARB enter the aquatic environment^9^. This is most critical because the wide spread of ARGs in allochthonous bacteria and horizontal gene transfer events can promote clinically relevant antibiotic resistance in pathogenic bacteria^10^.

Todays’ patients often already have ARB without any prior drug treatment, meaning that these bacteria must have acquired ARGs from environmental sources via horizontal gene transfer. In addition, the diversity of ARG variants has increased thereby adding to the ARG pool. For example, more than 1,000 different variants of β-lactamases have been described until today, which is a direct result of one of the most prescribed antibiotic drugs worldwide^11^. In addition, the accumulation of ARGs, promoting multi-resistant pathogens, makes antibiotic treatment especially difficult. This development is particularly dangerous in clinical settings, within the repetitive antibiotic application and insufficient hygiene enhancing the dissemination of ARB. The more problematic and hard-to-treat infections are caused by multi-resistant *Staphylococcus aureus* (MRSA), extended-spectrum β-lactamase (ESBL)-producing *Enterobacteriaceae* (or ESBL-producing *E. coli*), vancomycin-resistant enterococci (VRE), fluoroquinolone-resistant *Pseudomonas aeruginosa* (FQRP), and the upcoming resistant *Acinetobacter baumannii* and *Klebsiella pneumoniae* strains. All of the aforementioned organisms, genera, or families are normal residents of the healthy human or animal microbiome that can become opportunistic pathogens. They are therefore also referred to as FPB. A more extended list of problematic strains and resistance genes is studied in the GERMAP 2015^12^.

The aquatic environment is an important system for the development and dissemination of AMR. It represents an indefinite source of resistance genes, many of which are still unknown to us^13,14^. Therefore, countermeasures are needed to interrupt the ongoing ARG/ARB dissemination. Because, apart from the intrinsic fractions of ARGs, a high diversity of clinically relevant ARGs from hospital and communal wastewaters are additionally released to the aquatic environment day by day, and the recruitment of known and unknown ARGs into clinically relevant pathogens is a real threat^15^.

This study calculates the daily discharges of different categorized ARGs and FPB in effluents released from different communal WWTP clusters. These WWTPs differed in sizes and catchment areas with animal farming, food production, hospitals, and housing areas. Beside the overall discharge calculations, target-specific Pearson correlations were performed to identify a possible impact of the different catchment sites. To our knowledge, this is the first study evaluating an increased number of WWTP effluents with different catchment areas in 12 clinically relevant categories of ARGs and five FPB according to their daily discharge quantities and qualities.

## Results and Discussion

### ARGs and FPB daily discharges by different WWTP effluents

**Figure 1:**
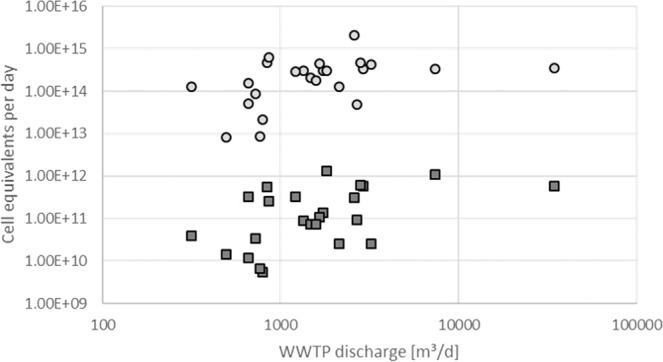
Daily discharge of the total amount of investigated ARGs (circles) and FPB (squares) by differently sized WWTPs (n = 23). Each circle and square represent an average value of four independent sampling periods during 2018. Deviations are listed in SI Tables 1–3.

To analyze the amount of ARGs and FPB discharged on a daily basis by each investigated WWTP, a sum of all detected ARGs and taxonomic marker genes for each WWTP was calculated and brought in relation to the corresponding daily WWTP effluent volume. Furthermore, this study focused on the detection of living FPB and ARGs of intact bacteria by excluding free DNA and ARG from injured or dead bacteria by living/dead discrimination to emphasize on the possible risk of subsequent human colonization by contact with these waters released to the environment. This means that the total daily discharge, taking ARGs from injured bacteria and free DNA into account, can be assumed to be considerably higher. Due to the use of reference bacteria carrying the respective antibiotic resistance gene for calculation of the abundances of ARB derived from genomic DNA of microbial communities in the water samples, the term “cell equivalents” is used. (Fig. 1).

### Antibiotic resistance of bacteria (ARG cell equivalents)

The average daily discharge across all WWTPs was calculated to be 3.30 × 10^14^ cell equivalents. Each individual WWTP cluster scored an average of 2.85 × 10^14^ (cluster C), 2.06 × 10^14^ (cluster F), and 4.99 × 10^14^ (cluster H) ARG cell equivalents per day. The biggest share of the ARGs sum parameter consisted of ARGs against macrolides (*erm*B), tetracycline (*tet*M), β-Lactame (*bla*_TEM_), and sulfonamide (*sul*1) representing 99.8–99.9% of the total ARG cell equivalent daily discharge and can therefore, as previously described by Hembach *et al*.^8^, be categorized as being “frequently abundant” in wastewater.

The β-lactame resistance genes *bla*_CMY2_, *bla*_CTX-M15_, *bla*_CTX-M32_, and the carbapeneme resistance gene *bla*_OXA48_, conveying enzymes against antibiotics of last resort in multi-resistant Gram-negative bacteria were categorized based on their abundance in the studied WWTP effluents as being “intermediately frequent”. This category represents 0.03–0.17% of the total ARG cell equivalent daily discharge.

The third category consisting of antibiotic resistance genes against antibiotics of last resort (carbapeneme resistance *bla*_NDM-1_, vancomycin resistance *van*A, methicilin resistance *mec*A, colistin resistance *mcr*-1) are considered to be “rarely occurring” in wastewaters. This category represents only 0.0001–0.00016% of the total ARG cell equivalent daily discharge. Most of the antibiotic resistance genes of the last two categories are located on mobile genetic elements^7,8^.

The highest average ARG cell equivalent discharge of all WWTPs was found in H1, which conditions wastewaters from a hospital and a catchment area of 34,000 PE (2.10 × 10^15^ cell equivalents per day, 2,584 m³ per day). The lowest calculated discharge of ARG cell equivalents belongs to C11, a communal WWTP with 8,000 PE (2.17 × 10^13^ cell equivalents per day, 495 m³ per day). In spite of higher effluent volumes of larger WWTPs, like H8 with 34,306 m^3^ per day, and 3.50 × 10^14^ ARG cell equivalents per day, bigger WWTPs did not necessarily demonstrate a higher emission of ARG cell equivalents in comparison to smaller WWTPs. For example, C7 with only 841 m³ per day displayed a comparable total daily ARG discharge of 4.59 × 10^14^ cell equivalents (SI Table 1).

While cluster H displayed the highest average ARG cell equivalent discharge of all clusters, only one WWTP of this cluster (i.e. H1) exhibited a significant elevated ARG emission (2.10 × 10^15^ cell equivalents per day) compared to the other WWTPs of all clusters (SI Table 3) (p < 0.05).

Cluster F consists of WWTPs with catchment areas comprising of population equivalents between 23,500 and 45,000 and ranks therefore in size between the purely communal WWTP cluster C and the hospital-influenced cluster H. The average ARG daily discharge in cluster F was calculated to be 2.06 × 10^14^ cell equivalents and is therefore below the average ARG cell equivalent daily discharge of clusters C and H by 27.8% and 57.7%, respectively. The effluent F3 (46,000 PE) exhibited the highest load of ARGs with 4.71 × 10^14^ cell equivalents per day within this cluster (impacted by a pig slaughter house). The lowest discharge of ARGs was analyzed in effluent F2 (23,500 PE, 767 m³ per day) with 8.56 × 10^12^ cell equivalents per day, treating wastewater from a cattle slaughter house. In consensus with clusters C and H, the daily discharge of ARG cell equivalents is not immediately tied to the WWTP size. This is also demonstrated by F4 (45,000 PE) and F1 (26,000 PE) with 4.76 × 10^13^ and 2.97 × 10^14^ ARG cell equivalents per day, respectively (SI Table 2).

Based on the numbers of total ARG cell equivalents discharge per day, it became obvious that WWTP size, independent of the catchment area, is not the main driving force for a high ARG dissemination. Smaller WWTPs are capable of releasing as many ARG cell equivalents per day as larger WWTPs, leading to the question as to what kind of wastewater treatment or treatment efficiency smaller WWTPs apply. The total ARG cell equivalents discharge of WWTPs with effluents less than 3,000 m³ per day display a deviation of more than two orders of magnitude.

**Figure 2:**
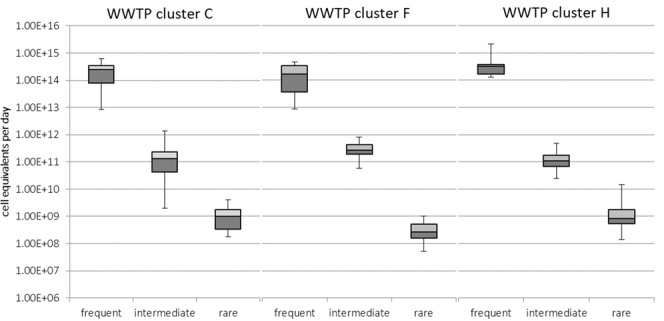
Abundance of frequent (*sul*1, *erm*B, *bla*_TEM_, *tet*M), intermediate (*bla*_CTX-M32_, *bla*_OXA48_, *bla*_CTX-M15_, *bla*_CMY-2_), and rare (*mec*A, *bla*_NDM-1_, *mcr*-1, *van*A) antibiotic resistance genes in cluster C (only communal WWTP effluents), cluster F (food production-impacted WWTP effluents), and cluster H (hospital-impacted WWTP effluents) displayed as cell equivalents per daily WWTP discharge volume. Each WWTP was sampled four times, and the significance was determined using the two-tailed non-parametric Mann-Whitney U test. Edges between two different bars represent the median. The bars themselves represent the upper (p = 0.75) and the lower (p = 0.25) quantiles. Error bars illustrate the maximum and minimum abundances.

While the daily discharges of ARG cell equivalents by WWTP effluents are mostly impacted by resistance genes of the category of “frequently abundant” ARGs, the distribution of the category “intermediately abundant” ARGs (*bla*_CTX-M32_, *bla*_CTX-M15_, *bla*_OXA48_, *bla*_CMY-2_) displaying a similar distribution within the different catchment areas (Fig. 2). The highest median daily ARG discharge value was calculated in cluster F with 2.69 × 10^11^ cell equivalents followed by clusters C and H with 1.31 × 10^11^ (51% less) and 1.07 × 10^11^ (60% less) cell equivalents. It has to be noted that while median daily ARG discharge values were very similar, the scattering in cluster C amounted to almost three orders of magnitude with effluent C4 (17,000 PE) displaying only 1.98 × 10^9^ cell equivalents (98% less than the median value) and effluent C7 (16,000 PE) emitting 1.38 × 10^12^ cell equivalents (ten times more than the median value).

ARGs of the category of occurring “rarely” (*mcr*-1, *van*A, *bla*_NDM-1_, *mec*A) exhibited similar median daily discharges within clusters C (9.78 × 10^8^ cell equivalents), F (2.61 × 10^8^ cell equivalents), and H (6.77 × 10^8^ cell equivalents) as well, but the impact of hospital wastewater becomes apparent comparing the maximum daily discharge values of each cluster. Here, the effluent of H2 displayed 1.42 × 10^10^ cell equivalents in contrast to the maximum of cluster C with 4.10 × 10^9^ (71% less) and cluster F with 1.0 × 10^9^ (93% less) ARG cell equivalents per day (see SI Tables 1–3).

### Facultative pathogenic bacteria

**Figure 3:**
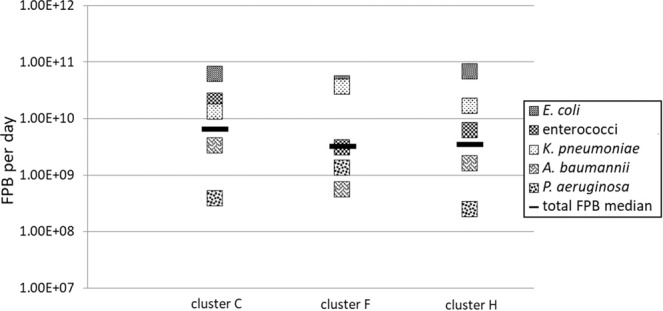
Daily discharge of facultative pathogenic bacteria in C) communal WWTP effluents (n = 11), F) food-producing impacted WWTP effluents (n = 4), and H) hospital-impacted WWTP effluents (n = 8). Displayed are the median values of each individual facultative pathogenic bacterium, as well as the median values of the total FPB for each WWTP effluent cluster. Deviations are listed in SI Tables 1–3.

In addition to considering ARG categories, selected FPB daily discharges were calculated using species-specific gene markers to quantify *E. coli*, *P. aeruginosa*, *K. pneumoniae*, *A. baumannii*, and enterococci. Furthermore, the total amounts of FPB daily discharge values of each WWTP were calculated (Fig. 3). In general, all WWTP effluents contained all five targeted FPB with median discharge values of up to three orders of magnitude below the ARGs’ median values, but still reaching cell equivalents per day of up to 1.34 × 10^12^ (C4, 17,000 PE) (see also SI Table 1). It has to be mentioned that a direct comparison of the ARG cell equivalent discharge values with FPB discharge values might be critical due to the lower numbers of analyzed FPB targets. In addition, the higher occurrence of genetic mobile ARGs in more than one bacterial species in the entire wastewater community might contribute to these differences in cell equivalent numbers.

The lowest abundance of FPB was calculated in the effluent water from C11 (26,300 PE) with 5.42 × 10^9^ cell equivalents per day. In agreement with the ARG daily discharge values, large WWTPs like H3 and H8 with 1.07 × 10^12^ and 5.85 × 10^11^ cell equivalents per day displayed similar FPB discharge values compared to smaller WWTPs (C7, 5.6 × 10^11^ cell equivalents per day, 16,000 PE). Similar to the ARG analyses, small WWTPs are analyzed to release as many FPB per day as larger WWTPs. Also, WWTPs with effluent volumes of less than 3,000 m³ per day display an FPB daily discharge deviation of more than two orders of magnitude.

Taxonomic specific analyses (Fig. 3) revealed a similar distribution of FPB in the effluents of clusters C and H with a 3.3 times more dominant enterococci abundance in cluster C (2.03 × 10^10^ cell equivalents per day) compared to cluster H (6.05 × 10^9^ cell equivalents per day). In both clusters, *E. coli* exhibited by far the highest daily discharge values of all investigated FPB accounting for 62% (cluster C) and 73.6% (cluster H) of total FPB daily discharges. In contrast, effluent cluster F displayed a different FPB distribution. Here, *E. coli* reached a similar 50% share of total FPB daily discharge values, but the *K. pneumoniae* share was up to 2.8 and 2.2 times higher compared to the shares found in clusters C and H, reaching 44% of the total FPB daily discharge of cluster F. Also, *P. aeruginosa* was more abundant in cluster F reaching daily discharges of 1.32 × 10^9^ cell equivalents compared to clusters C (3.78 × 10^8^ cell equivalents per day) and H (2.44 × 10^8^ cell equivalents per day). These findings demonstrate the impact on the FPB distribution in wastewater derived primarily from humans (clusters C and H) compared to wastewater impacted by animal sewages. Nevertheless, the median daily discharge values summarizing all of the five FPB mentioned ranged from 10^9^ to 10^10^ cell equivalents per day in all WWTP clusters.

### Calculation of the Pearson coefficient of single bacterial gene targets from different WWTPs

It became obvious that the summarized median values of ARGs and FPB cell equivalents comparing the three clusters of WWTP effluents, especially hospital-impacted and non-hospital impacted WWTP effluents, revealed no significant difference in the bacteria and ARG target qualities and quantities (p > 0.05). It has to be mentioned that the percentage of hospital wastewaters in the total volumes of the WWTP streams ranged only between 0.43 and 1.73 vol%. Therefore, Pearson correlations of the individual bacterial parameters among the different clusters of WWTPs (communal, hospital-impacted, and food processing-impacted) were performed. In contrast to the daily discharge calculations, the Pearson coefficient is not based on sum parameters of ARG or FPB cell equivalents, but on absolute concentrations of each single ARG and taxonomy marker gene, which was quantified for each sampling period expressed as cell equivalent per 100 mL.

In agreement with the daily discharge results, no strong correlation was exhibited between the analyzed bacterial parameters (ARG, FPB) and the sizes of the different WWTPs (Fig. 1). However, in detail, a difference between hospital- and non-hospital-impacted WWTP effluents was observed for the individual parameter *E. coli*. This bacterium correlated in communal, non-hospital-impacted WWTP effluents (cluster C) with the frequently occurring ARGs *sul*1 (+0.701), *erm*B (+0.859), and *tet*M (+0.889), with the intermediately abundant *bla*_CTX-M32_ (+0.783), *bla*_CTX-M15_ (+0.971), and *bla*_CMY2_ (+0.755), and with the rarely occuring *mcr*-1 (+0.538). In contrast, however, *E. coli* correlated more distinctly with the highly critical carbapeneme resistance gene *bla*_NDM-1_ (+0.397) in hospital-impacted WWTP effluents of cluster H, but demonstrated lower correlation coefficients to other ARGs. The abundance of the frequently occuring ARGs was also recently described in *E. coli* isolates from patients^16,17^, but also from environmental samples^18^. Similar changes in correlation profiling between effluents of clusters H and C were also observed for other FPB like *K. pneumoniae*, *A. baumannii*, *P. aeruginosa*, and enterococci (Table 1). The abundance of carbapenem resistance genes especially in Gram-negative bacteria from hospital-influenced wastewater was also previously described^19^, and was mentioned by the WHO to be one of the most important challenges in modern medicine^1^. Another reason for the increased abundance of carbapeneme resistance in FPB in hospital wastewaters can be attributed to an antibiotic reservoir phenomenon in the drainpipes in clinics^20^. Here, the persistance of high concentrations of antibiotics in biofilms is documented to promote the selection of carbapeneme-resistant FPB. Interestingly, enterococci demonstrated an additional correlation with the vancomycin resistance gene *van*A in effluents of cluster H, which mediates a resistance against this glycopeptide antibiotic of last choice in the case of enterococci infections. This correlation between enterococci and *van*A (+0.713) together with the lower correlation between *mcr*-1 and *E. coli* (+0.278) was also observed in cluster F. These were the only differences between clusters C and F (data not shown).

**Table 1:** Pearson correlation coefficient of facultative pathogenic bacteria and antibiotic resistance genes in treated wastewater with no influence of hospital wastewater (WWTP cluster C) and with hospital-influenced wastewater (WWTP cluster H).

	sulfonamid resistance (*sul*1)	erythromycin resistance (*erm*B)	β-lactam resistance (*bla*_TEM_)	tetracycline resistance (*tet*M)	cephalosporine resistance (*bla*_CTX-M32_)	carbapenem resistance (*bla*_OXA48_)	cephalosporine resistance (*bla*_CTX-M15_)	ampicillin resistance (*bla*_CMY-2_)	methicillin resistance (*mec*A)	carbapenem resistance (*bla*_NDM-1_)	colistin resistance (*mcr*-1)	vancomycin resistance (*van*A)	mobile genetic element (*intI*1)
**WWTP effluents of cluster C**													
*E. coli*	**0.701**	**0.859**	-0.098	**0.889**	**0.783**	0.431	**0.971**	**0.755**	0.484	0.001	**0.538**	0.233	0.211
*K. pneumoniae*	0.365	0.443	0.018	**0.735**	0.195	**0.659**	**0.717**	0.460	0.206	0.052	0.252	0.164	0.333
enterococci	0.356	0.342	−0.114	**0.616**	0.174	0.374	**0.538**	0.308	0.245	0.256	0.151	0.157	0.179
*A. baumannii*	**0.649**	**0.730**	−0.001	**0.734**	**0.499**	0.461	**0.811**	**0.622**	0.433	0.112	0.455	0.459	0.178
*P. aeruginosa*	**0.615**	**0.695**	−0.152	**0.861**	**0.575**	**0.531**	**0.600**	**0.600**	0.372	0.024	0.496	**0.573**	0.464
**WWTP effluents of cluster H**													
*E. coli*	0.152	0.216	−0.080	0.081	0.203	0,138	0.267	0.051	−0.026	0.395	0.161	−0.058	0.067
*K. pneumoniae*	0.046	**0.702**	0.118	0.415	**0.674**	0,148	**0.775**	0.403	0.128	**0.766**	0.123	0.271	0.218
*enterococci*	0.085	**0.705**	0.195	0.298	**0.719**	0,364	**0.825**	0.321	0.365	**0.783**	0.143	**0.718**	**0.601**
*A. baumannii*	0.168	**0.598**	0.027	0.214	0.479	**0,549**	**0.536**	0.348	**0.588**	**0.723**	0.352	0.027	**0.667**
*P. aeruginosa*	0.167	0.426	−0.098	0.206	0.358	**0,679**	**0.557**	0.279	**0.623**	**0.815**	0.263	−0.077	**0.896**

A Pearson correlation coefficient >0.1 corresponds to a low/weak correlation, a coefficient >0.3 corresponds to a medium/moderate correlation, and a coefficient >0.5 (bold) corresponds to a strong/significant correlation between two parameters^38,39^. A Pearson correlation coefficient <0 corresponds to a counter/inverse correlation.

On the other hand, *sul*1 and *tet*M have a higher Pearson correlation with FPB in effluents of cluster C compared to cluster H, reaching up to +0.701 for *sul*1 and +0.889 for *tet*M. The same bacterial taxonomy markers exhibited only weak correlations with *sul*1 (max. +0.168) or *tet*M (max. +0.415) in hospital-impacted wastewater effluents of cluster H. One reason for these disparities in correlation profiles might be the different antibiotic prescriptions in outpatient care compared to inpatient treatments.

In fact, the GERMAP 2015 report indicated sulfonamide and tetracyclin antibiotics to be more frequently prescribed in outpatient care than in hospital treatments, where tetracycline and sulfonamide antibiotics ranked 12^th^ and 10^th^ of the most frequently prescribed classes of antibiotics, in contrast to outpatient care. There, the same antibiotics are ranked 4^th^ and 9^th^ of the most frequently prescribed antibiotics. In addition, sulfonamides are not prescribed seperatley, but are frequently used in combination with other drugs for local applications (70 Mio. DDD) or urinary tract infections (13.6 Mio. DDD). Considering this, sulfonamides belong to the most frequently prescribed antibiotics in outpatient care.

In addition, methods like microbial source tracking could help to indentify the origin behind higher discharge concentrations of ARGs/FPB in corresponding WWTP effluents (e. g. H1). The WWTP treatment setups investigated here were very similar in design, differing only in small changes to the primary and secondary treatments, which is unlikely to be the main reason for the high variation in the daily discharged ARGs and FPB especially of small WWTPs. This aspect is important when considering centralized/decentralized actions to decrease the ARGs/FPB discharge amount of critical identified WWTPs. The treatment of low-volume, but highly contaminated hospital wastewater is in most cases more cost efficient compared to the higher volumes in communal wastewater treatments. In addition, communal or industrial wastewaters also contribute to antibiotic resistance loads of sewer systems reaching the WWTPs and the subsequent receiving waters being connected to subjects of specific protection (i.e. bathing waters, drinking water resources, agricultural field irrigation). Hence, to treat the combined wastewaters at the communal WWTP might be more advisable considering the possible health risk in the case of colonizing humans and/or possible infections with resistant and multiresistant bacteria^21^.

Furthermore, Pearson coefficient calculation demonstrated for effluent cluster H that the class-1 integron-specific *intl*1 gene, an important factor in the horizontal gene transfer (HGT) of, e.g. genetically mobile antibiotic resistance genes among bacteria in the environment^22^, corresponded positively with FPB: *A. baumannii* (+0.667), *P. aeruginosa* (+0.896), and enterococci (+0.601). This is not the case in wastewater effluents of cluster C, where only weak (+0.178 and +0.179) or moderate correlations (+0.464) became visible between the occurrence of *intl*1 gene and marker genes of FPB. Dispite the known issues of Pearson coefficient correlation statistics^23^, these calculations indicated a distinct quality difference in ARG carriers of conditioned wastewater from hospital-impacted WWTPs, while an absolute quantification indicated no significant difference between ARG cell equivalent discharges from WWTPs with different catchment areas.

In addition, Paulus *et al*.^24^ demonstrated an up to 2.3 fold increased prevalence of ARGs in on-site hospital wastewaters compared to communal wastewaters, but the spectrum of ARGs was only reduced by the lack of *bla*_KPC_ and *van*A antibiotic resistance genes coding for carbapeme and vancomycin resistance in the final communal WWTP effluent. Furthermore, it was suggested that an on-site treatment of hospital wastewater would significantly reduce the ARG burden in subsequent WWTPs. While we recognize the benefit of an effective on-site treatment of wastewater with significant hospital sewage percentage, quantitative analysis demonstrated comparable total ARG concentrations originating from communal as well as food-processing wastewaters. However, also carbapeneme, colistin, and vancomycin resistance genes (*bla*_OXA48_, *bla*_NDM-1_, *mcr*-1, *van*A) were present in the effluents of all three WWTP clusters including housing areas. While in this study, only the final WWTP effluents were analyzed (no before-after wastewater treatment comparison), the question arises as to which quantitative threshold of hospital wastewater determines a significant impact on the final WWTP effluent quality with regard to multi-resistant bacteria and antibiotics bearing a potential for selection of ARB even at sub-inhibitory concentrations^25^ (polluter-pays principles). Also, how important is the influence of the efficiency of conventional wastewater treatment on the reduction of ARGs and FPB, when one considers the high variations in the final effluent of small WWTPs below a daily discharge volume of 3,000 m³. Here, advanced treatment technologies at communal WWTPs should be considered. Hembach *et al*.^8^ described an effective combination of advanced technologies directing the elimination of ARGs and FPB at a real WWTP.

## Conclusion

This study gives an insight into the daily discharge of ARGs and FPB by differently sized WWTPs in Germany with different catchment areas. Absolute quantification proved that the complete spectrum of antibiotic resistance genes and facultative pathogenic bacterial targets passed the WWTPs and were released with the effluents to the environments independently from the sizes and daily wastewater discharge volumes of the investigated treatment plants. While the discharged quantities of the investigated ARGs and FPB between the different WWTP clusters were comparable, correlation analysis (PC) of hospital-influenced WWTPs showed an increased abundance of clinically relevant ARGs to be associated with distinct FPB. Nevertheless, all WWTP effluents contributed to the broad spectrum of ARGs and FPB released to the receiving compartments. In consequence, the receiving waters bear a possible health risk by colonizing humans with these bacteria. Here, specific protection areas should be defined to avoid a possible contamination of water resources being important to health surveillance (i.e. bathing waters, drinking water resources, agriculture field irrigation). Especially the further dissemination of detected and exceptionally critical ARGs including resistance against carbapeneme, vancomycin, and colistin in facultative pathogenic bacteria (e.g. ESKAPE group) should be stopped, because of the importance of these antibiotics in modern medicine. As stated by the WHO, there are almost no alternative treatment options in the case of patient´s infections with these antibiotic-resistant and multi-resistant bacteria. This study drastically supports the important need of advanced combined treatment systems (e.g. membrane technologies, oxidative treatment) to stop the dissemination of antibiotic-resistant genes in facultative pathogenic bacteria as recommend by the WHO, UN assembly, and G7 summit. Since communal WWTPs are the direct link to the aquatic environment, WWTPs should be monitored according to their ARG and FPB abundances and discharges to decide about the need of advanced treatment options. Here, critical threshold volumes of hospital wastewaters should be defined to discuss the effect of a decentralized wastewater treatment.

## Material and Methods

### Wastewater treatment plants and bacterial targets directing antibiotic resistance genes or facultative pathogenic bacterial gene markers

Aiming at the calculation of daily discharges of ARB/ARGs and selected FPB, a total number of 23 conventionally treated WWTP effluents were investigated over four sampling campaigns between February and November (Table 2). A total of 92 WWTP effluents were analyzed for the abundance of 12 ARGs, 5 FPB, and one mobile genetic element. The conventional treatmet of the raw wastewaters consisted of a mechanical treatment, followed by activated sludge-mediated biological treatment, and a final clarification step (SI Table 5). 11 WWTPs treat wastewater from housing areas alone and are grouped into cluster C (communal), while four additional WWTPs condition different volumes of wastewaters from animal and food processing companies (cluster F, food-production). The last eight WWTPs were impacted by hospital wastewaters between 0.43 vol% and 1.73 vol% and are grouped into cluster H (hospital). A specific overview of WTP treatment setups is displayed in SI Table 5.

**Table 2:** List of wastewater treatment plants under investigation.

WWTP cluster	WWTP Acronym	Population equivalents	sampling	catchment area
communal (C)	C1	11,000	grab samples	with wastewater from regeneration hospitals
	C2	13,500	grab samples	communal/housing wastewater
	C3	14,000	grab samples	communal/housing wastewater
	C4	17,000	grab samples	communal/housing wastewater
	C5	16,600	grab samples	communal/housing wastewater
	C6	16,150	grab samples	communal/housing wastewater
	C7	16,000	grab samples	communal/housing wastewater
	C8	26,300	grab samples	communal/housing wastewater
	C9	10,000	grab samples	communal/housing wastewater
	C10	7,200	grab samples	communal/housing wastewater
	C11	8,000	24 h composite	communal/housing wastewater
food (F)	F1	26,000	grab samples	with dairy wastewater
	F2	23,500	24 h composite	with slaughter house wastewater (cattle)
	F3	46,000	24 h composite	with slaughter house wastewater (pork)
	F4	45,000	24 h composite	with slaughter house wastewater (chicken)
hospital (H)	H1	34,000	grab samples	with 0.71 vol% hospital wastewater
	H2	21,500	grab samples	with 1.16 vol% hospital wastewater
	H3	58,000	grab samples	with 0.63 vol% hospital wastewater
	H4	27,000	grab samples	with 1.73 vol% hospital wastewater
	H5	43,000	grab samples	with 1.64 vol% hospital wastewater
	H6	15,000	grab samples	with 0.43 vol% hospital wastewater
	H7	13,000	grab samples	with 0.56 vol% surgery wastewater
	H8	210,000	24 h composite	with 1.16 vol% hospital wastewater

The sizes of the WWTPs correspond with the population equivalent values. The different WWTPs possess different catchment areas including hospitals, food processing companies, or housing areas only.

### Sampling, sample preparation, and DNA extraction

Four sampling campaigns were performed during the year 2018 (February until November). Volumes of 200 mL of each individual effluent water were vacuum-filtrated through a 0.2 µm polycarbonate membrane (Whatman Nuclepore Track-Etched Membranes, Sigma-Aldrich, Munich, Germany). After the filtration process, the membranes were treated with 0.25 mM propidium monoazide (BLU-V viability PMA-kit, Quiagen GmbH, Hilden, Germany) to complex free DNA as well as DNA from dead or injured bacteria^26^. DNA extraction from the filtered biomass from the membrane was performed using the Fast DNA Spin Kit for Soil and FASTPREP Instrument (MP Biomedicals, Santa Ana, CA) according to previous studies and stored until further analysis at −20 °C^7^.

### Taxonomic gene markers and antibiotic resistance genes

All qPCR systems targeting ARGs and marker genes specific for FPB were run in technical triplicates according to Hembach *et al*. 2017 and 2019^7,8^.The average discharge concentration for each investigated parameter and WWTP as well as full primer sequences are listed in the Supplementary Information (SI Tables 1,2,3, and 4). *Klebsiella pneumoniae* (*gltA* gene)^27^, *Pseudomonas aeruginosa* (*ecfX* gene)^27^*, Escherichia coli* (*yccT* gene)^27^, *Acinetobacter baumannii* (*secE* gene)^27^, and enterococci (23 S rRNA gene)^28^ were quantified. The antibiotic resistance genes *sul*1 (sulfamethoxazole resistance)^29^, *erm*B (erythromycin resistance)^30^, *bla*_TEM_ (β-lactam resistance)^29^, and *tet*M (tetracycline resistance)^31^, were studied as they represent the most frequently detected ARGs in urban wastewaters^8^. The antibiotic resistance genes *bla*_CTX-M-15_^32^, *bla*_CTX-M-32_^29^, *bla*_OXA-48_ (carbapeneme resistances)^33^, and *bla*_CMY-2_ (ampicillin resistance)^34^, were also analyzed. Furthermore, the resistance genes *mec*A (methicillin resistance in staphylococci)^35^, *van*A (vancomycine resistance)^36^, *mcr*-1 (colistin resistance)^7^, and *bla*_NDM-1_ (new deli-β-lactamase)^33^ were monitored as they represent resistances against last-line-of-defence antibiotics.

For qPCR analysis, a Bio-Rad Cycler CFX96 (CFX96 Touch Deep Well Real-Time PCR Detection System, Bio-Rad, Munich, Germany) was used. All samples were measured in technical triplicates. The specific taxonomic and resistance-directed genes in the entire bacterial community were quantified utilizing an SYBR Green qPCR approach according to Hembach *et al*., and Jäger *et al*.^7,37^. Cell equivalent calculation according to Hembach *et al*.^7,8^ was performed to calculate the absolute concentration of ARG/FPB normalized to 100 mL of water sample.

### Pearson’s correlation

Pearson’s correlation coefficient was applied to a series of measurements (SI Table 1–3) using a pairing of each taxonomic parameter (*x*_*i*_) to all consecutive antibiotic resistance genes (*y*_*i*_) and is represented by *r*_*xy*_. Due to the pairing of the data, the sample correlation coefficient ${r}_{xy}$ is defined as:

$${r}_{xy}:=\frac{{\sum }_{i=1}^{n}({x}_{i}-\bar{x})({y}_{i}-\bar{y})}{\sqrt{{\sum }_{i=1}^{n}{({x}_{i}-\bar{x})}^{2}}\cdot \sqrt{{\sum }_{i=1}^{n}{({y}_{i}-\bar{y})}^{2}}}$$

Where *n* is the sample size, *x*_*i*_ and *y*_*i*_ the individual taxonomic and antibiotic resistance gene values indexed with *i*, as well as $\bar{x}=\frac{1}{n}{\sum }_{i=1}^{n}{x}_{i}$ (the parameter mean value); and analogously for $\,\bar{y}$.

The sample correlation coefficient ranges from −1 to 1. A value of 1 implies that a linear equation describes the relationship between *x* and *y* perfectly, with all data points lying on a line for which *y* increases as x increases. A value of −1 implies that all data points lie on a line for which y decreases as x increases. A value of 0 implies that there is no linear correlation between the variables.

## Supplementary information

Supplemental information.
