# Supplementary material for: Evaluation of antibiotic resistance dissemination by wastewater treatment plant effluents with different catchment areas in Germany
Source: Sci Rep. 2020 Jun 2;10:8952. doi: 10.1038/s41598-020-65635-4 (PMC7265433; doi:10.1038/s41598-020-65635-4)
Supplement: Supplementary file 1 — Supplemental information. [file 41598_2020_65635_MOESM1_ESM.pdf]

Supplementary Information

"**Evaluation of antibiotic resistance dissemination by wastewater treatment plant effluents with different catchment areas in Germany**" with the authors Johannes Alexander, Norman Hembach, and Thomas Schwartz.

Number of pages: 8

Number of tables 5

Corresponding authors:

Dr.-Ing. Johannes Alexander
Karlsruhe Institute of Technology (KIT), Institute of Functional Interfaces (IFG),
Microbiology Molecular Biology Department
Herrmann von Helmholtz Platz 1, 76344 Eggenstein-Leopoldshafen, Germany
E-Mail: johannes.alexander@kit.edu; phone: +49 721 608-26802; fax: +49 721 608-26858.

SI Table 1: Daily discharge of ARG and FPB cell equivalents by communal WWTPs. Displayed are the average discharge concentration for each investigated parameter per day and the median value and deviation per group (frequently, intermediately, rarely occurring, and bacteria). Each WWTP was samples 4 times.

**WWTP discharge [m³/d]**	841	1,730	1,647	860	660	1,216	720	314	1,481	1,825	495	median of summation	deviation	
**WWTP acronym**	C7	C2	C5	C8	C1	C3	C9	C10	C6	C4	C11		min.	max.
**antibiotic resistance gene [cell equivalents/d]**														
sulfonamid resistance (*sul*1)	2.60E+14	2.63E+14	3.43E+14	2.43E+14	2.72E+13	6.29E+13	2.19E+13	1.91E+13	1.49E+14	1.60E+14	7.39E+12			
erythromycin resistance (*erm*B)	1.24E+14	3.21E+13	8.57E+13	3.00E+13	2.31E+13	2.84E+13	8.07E+12	6.86E+12	1.56E+13	9.17E+13	8.79E+11			
ß-lactam resistance (*bla*$_{TEM}$)	7.25E+13	1.17E+13	1.79E+13	3.43E+14	3.90E+11	1.91E+14	5.66E+13	1.01E+14	3.93E+13	4.32E+13	1.34E+13			
tetracycline resistance (*tet*M)	2.26E+12	1.20E+12	7.93E+11	5.45E+11	8.20E+11	7.63E+11	6.37E+10	4.31E+10	3.16E+11	3.04E+12	1.63E+10			
summation frequently abundant ARG	4.59E+14	3.08E+14	4.47E+14	6.17E+14	5.15E+13	2.83E+14	8.66E+13	1.27E+14	2.04E+14	2.98E+14	2.17E+13	2.83E+14	2.17E+13	6.17E+14
cephalosporine resistance (*bla*$_{CTXM32}$)	1.31E+12	3.10E+10	1.01E+11	5.49E+10	2.24E+10	1.00E+11	2.52E+10	3.61E+04	1.51E+11	1.18E+11	1.78E+09			
carbapenem resistance (*bla*$_{OXA48}$)	4.95E+10	1.89E+11	1.01E+11	2.11E+09	7.16E+10	5.60E+10	1.92E+10	6.77E+04	7.38E+10	8.32E+11	1.00E+00			
cephalosporine resistance (*bla*$_{CTX-M15}$)	2.37E+10	3.83E+09	2.45E+09	3.76E+09	2.00E+09	6.87E+09	6.02E+08	2.27E+03	1.13E+10	4.36E+10	1.94E+08			
ß-lactam resistance (*bla*$_{CMY-2}$)	3.49E+09	1.23E+09	5.16E+08	1.34E+09	1.36E+09	8.72E+08	5.76E+08	2.27E+02	6.75E+08	2.83E+09	1.00E+00			
summation intermediate abundant ARG	1.38E+12	2.26E+11	2.05E+11	6.21E+10	9.74E+10	1.64E+11	4.56E+10	1.06E+05	2.37E+11	9.97E+11	1.98E+09	1.64E+11	1.06E+05	1.38E+12
methicillin resistance (*mec*A)	9.00E+08	2.23E+08	6.62E+08	6.51E+07	1.31E+07	1.08E+08	7.49E+07	4.11E+07	2.13E+07	3.40E+09	9.58E+07			
carbapeneme resistance (*bla*$_{NDM-1}$)	4.26E+08	1.57E+08	3.80E+08	1.41E+09	3.38E+08	4.70E+08	1.00E+00	1.00E+00	1.90E+07	3.60E+08	8.21E+07			
colistin resistance (*mcr*-1)	5.82E+08	7.55E+08	8.29E+08	2.36E+08	2.66E+08	3.66E+08	2.68E+08	1.27E+08	1.34E+08	3.51E+08	0.00E+00			
vancomycin resistance (*van*A)	2.39E+08	2.32E+07	1.00E+00	1.00E+00	1.17E+08	1.28E+07	1.22E+07	1.06E+07	9.55E+07	1.00E+00	4.04E+07			
summation rarly occuring ARG	2.15E+09	1.16E+09	1.87E+09	1.71E+09	7.34E+08	9.57E+08	3.55E+08	1.79E+08	2.70E+08	4.11E+09	2.18E+08	9.57E+08	1.79E+08	4.11E+09

**facultative pathogenic bacteria [cell equivalents/d]**														
E. coli (*yccT*)	4.74E+11	1.11E+11	6.20E+10	5.81E+10	8.87E+10	7.33E+10	2.74E+10	2.33E+10	3.81E+10	8.12E+11	2.09E+09			
enterococci (*23SrRNA*)	5.11E+10	9.90E+09	1.98E+10	1.62E+11	1.59E+11	2.09E+11	3.94E+09	4.87E+09	2.08E+10	3.43E+11	2.75E+09			
K. pneumoniae (*gltA*)	2.90E+10	1.20E+10	1.38E+10	2.70E+10	6.91E+10	3.94E+10	1.77E+09	1.03E+10	9.07E+09	1.69E+11	4.70E+08			
A. baumannii (*secE*)	4.39E+09	2.50E+09	8.04E+09	5.56E+09	5.41E+09	2.73E+09	3.19E+08	4.43E+08	3.82E+09	1.16E+10	1.14E+08			
P. aeruginosa (*ecfX*)	1.58E+09	6.01E+08	1.02E+09	3.86E+08	2.94E+08	3.70E+08	2.73E+08	2.90E+08	1.16E+09	3.71E+09	1.00E+00			
summation FPB	5.60E+11	1.36E+11	1.05E+11	2.53E+11	3.22E+11	3.24E+11	3.37E+10	3.92E+10	7.30E+10	1.34E+12	5.42E+09	1.36E+11	5.42E+09	1.34E+12

SI Table 2: Daily discharge of ARG and FPB cell equivalents by food production-impacted WWTPs. Displayed are the average discharge concentration for each parameter per day and the median value and deviation per group (frequently, intermediately, rarely occurring, and bacteria). Each WWTP was samples 4 times.

**WWTP discharge [m³/d]**	1,340	767	2,800	2,687	median of summation	deviation	
**WWTP acronym**	F1	F2	F3	F4		min.	max.
**antibiotic resistance gene [cell equivalents/d]**							
sulfonamid resistance (*sul*1)	2.21E+14	5.82E+12	4.57E+14	4.09E+13			
erythromycin resistance (*erm*B)	4.57E+13	1.40E+12	6.86E+12	1.40E+12			
ß-lactam resistance (*bla*TEM)	2.90E+13	1.26E+12	6.50E+12	4.88E+12			
tetracycline resistance (*tet*M)	2.97E+11	3.43E+10	2.56E+11	2.12E+11			
summation frequently abundant ARG	2.96E+14	8.51E+12	4.71E+14	4.74E+13	1.72E+14	8.51E+12	4.71E+14
cephalosporine resistance (bla_{CTXM32})	1.99E+11	4.87E+07	5.06E+09	1.20E+10			
carbapeneme resistance (bla_{OXA48})	6.08E+11	1.81E+06	3.03E+11	2.35E+11			
cephalosporine resistance ($bla_{CTX\text{-}M15}$)	4.48E+09	1.48E+06	4.87E+08	4.96E+10			
ß-lactam resistance ($bla_{CMY\text{-}2}$)	4.78E+09	0.00E+00	8.87E+05	8.52E+05			
summation intermediate abundant ARG	8.15E+11	5.20E+07	3.08E+11	2.97E+11	3.02E+11	5.20E+07	8.15E+11
methicillin resistance (*mec*A)	8.50E+08	4.87E+07	1.29E+08	3.38E+08			
carbapeneme resistance ($bla_{NDM\text{-}1}$)	0.00E+00	1.81E+06	4.72E+06	4.53E+06			
colistin resistance (*mcr*-1)	1.18E+08	1.48E+06	5.59E+07	3.70E+06			
vancomycin resistance (*van*A)	3.33E+07	0.00E+00	0.00E+00	0.00E+00			
summation rarly occuring ARG	1.00E+09	5.20E+07	1.89E+08	3.46E+08	2.68E+08	5.20E+07	1.00E+09

**facultative pathogenic bacteria [cell equivalents/d]**							
E. coli (*yccT*)	1.84E+10	4.70E+09	2.53E+11	6.37E+10			
enterococci (*23SrRNA*)	1.69E+10	1.73E+08	2.03E+09	4.05E+09			
K. pneumoniae (*gltA*)	4.92E+10	1.43E+09	3.51E+11	2.30E+10			
A. baumannii (*secE*)	3.81E+09	8.51E+07	8.20E+08	2.59E+08			
P. aeruginosa (*ecfX*)	1.28E+08	6.44E+07	2.52E+09	2.61E+09			
summation FPB	8.85E+10	6.45E+09	6.10E+11	9.36E+10	9.10E+10	6.45E+09	6.10E+11

SI Table 3: Daily discharge of ARG and FPB cell equivalents by hospital-impacted WWTPs. Displayed are the average discharge concentration for each parameter per day and the median value and deviations per group (frequently, intermediately, rarely occurring, and bacteria). Each WWTP was samples 4 times.

**WWTP discharge [m^3/d]**	2,895	2,583	1,581	662	2,136	3,240	7,421	34,306	median of summation	deviation	
**WWTP acronym**	H2	H1	H6	H7	H4	H5	H3	H8		min.	max.
**antibiotic resistance gene [cell equivalents/d]**											
sulfonamid resistance (*sul*1)	1.91E+14	1.22E+14	1.08E+14	1.43E+14	6.86E+13	1.20E+14	2.35E+14	1.17E+13			
erythromycin resistance (*erm*B)	1.07E+14	6.70E+13	1.18E+13	8.30E+12	1.81E+13	3.12E+13	2.66E+13	1.10E+13			
ß-lactam resistance (*bla*$_{TEM}$)	2.64E+13	1.91E+15	5.55E+13	3.75E+12	4.26E+13	2.69E+14	6.60E+13	3.27E+14			
tetracycline resistance (*tet*M)	1.99E+12	1.26E+12	2.28E+11	5.06E+10	5.10E+10	4.55E+12	1.63E+11	2.57E+11			
summation frequently abundant ARG	3.27E+14	2.10E+15	1.75E+14	1.55E+14	1.29E+14	4.25E+14	3.28E+14	3.50E+14	3.27E+14	1.29E+14	2.10E+15
cephalosporine resistance (*bla*$_{CTXM32}$)	2.34E+11	2.08E+11	4.79E+10	1.08E+10	4.72E+10	1.07E+11	4.01E+10	2.08E+10			
carbapeneme resistance (*bla*$_{OXA48}$)	2.34E+11	6.01E+10	5.48E+10	1.31E+10	1.32E+10	1.40E+10	1.09E+11	6.84E+10			
cephalosporine resistance (*bla*$_{CTX-M15}$)	1.96E+10	1.47E+10	1.24E+09	7.17E+08	4.32E+09	9.31E+09	4.45E+09	4.50E+09			
ß-lactam resistance (*bla*$_{CMY-2}$)	2.23E+09	1.39E+09	6.92E+08	1.00E+00	6.15E+08	3.79E+09	7.06E+08	8.46E+06			
summation intermediate abundant ARG	4.90E+11	2.84E+11	1.05E+11	2.46E+10	6.54E+10	1.34E+11	1.54E+11	9.38E+10	1.19E+11	2.46E+10	4.90E+11
methicillin resistance (*mec*A)	5.98E+08	2.56E+08	1.51E+08	5.85E+07	2.45E+08	3.65E+08	2.77E+07	2.98E+08			
carbapeneme resistance (*bla*$_{NDM-1}$)	1.20E+10	5.74E+08	1.87E+08	1.81E+07	5.02E+08	7.60E+07	4.39E+07	4.50E+07			
colistin resistance (*mcr*-1)	1.60E+09	3.94E+08	2.50E+09	5.99E+07	5.34E+06	1.66E+08	1.41E+08	5.53E+08			
vancomycin resistance (*van*A)	1.00E+00	9.79E+07	6.94E+07	1.00E+00	1.82E+07	2.66E+07	1.42E+07	0.00E+00			
summation rarly occuring ARG	1.42E+10	1.32E+09	2.90E+09	1.36E+08	7.71E+08	6.34E+08	2.27E+08	8.96E+08	8.33E+08	1.36E+08	1.42E+10

**facultative pathogenic bacteria [cell equivalents/d]**											
E. coli (*yccT*)	2.50E+11	7.47E+10	5.95E+10	7.28E+09	2.04E+10	5.39E+09	1.06E+12	2.76E+11			
enterococci (*23SrRNA*)	2.41E+11	1.72E+11	8.07E+09	1.40E+08	1.57E+09	4.04E+09	3.17E+08	1.20E+11			
K. pneumoniae (*gltA*)	7.93E+10	6.20E+10	3.80E+09	3.07E+09	2.02E+09	1.60E+10	1.65E+10	1.83E+11			
A. baumannii (*secE*)	1.69E+10	2.44E+09	1.89E+09	9.08E+08	1.26E+09	1.00E+00	1.00E+00	3.23E+09			
P. aeruginosa (*ecfX*)	2.18E+09	3.71E+08	1.73E+08	1.00E+00	3.15E+08	3.26E+07	1.55E+08	2.98E+09			
summation FPB	5.89E+11	3.12E+11	7.34E+10	1.14E+10	2.55E+10	2.55E+10	1.07E+12	5.85E+11	1.93E+11	1.14E+10	1.07E+12

SI Table 4: Full primer sequences of investigated ARGs and FPB with corresponding calibration curve, efficiency and LOD

target	primer sequence	calibration curve	amplicon	efficiency	R^2	LOD	reference strain	literature
**facultative pathogenic bacteria**								
enterococci	Fwd: AGAAATTCCAAACGAACTTG<br>Rev: CAGTGCTCTACCTCCATCATT	F(x)= -3.585x+35.283	93 bp	90.1 %	1.000	65	*E. faecium* DSM20477	(Frahm et al. 2003)
P. aeruginosa	Fwd: AGCGTTCGTCCTGCACAAGT<br>Rev: TCCACCATGCTCAGGGAGAT	F(x)= -3.282x+35.276	81 bp	101.7 %	0.999	3	*P. aeruginosa* DSM1117	(Clifford et al. 2012)
K. pneumoniae	Fwd: ACGGCCGAATATGACGAATTC<br>Rev: AGAGTGATCTGCTCATGAA	F(x)= -3.387x+38.844	68 bp	97.4 %	0.998	18	*K. pneumoniae* DSM30104	(Clifford et al. 2012)
A. baumannii	Fwd: GTTGTGGCTTTAGGTTTATTATACG<br>Rev: AAGTTACTCGACGCAATTCG	F(x)= -3.380x+35.679	94 bp	97.6 %	1.000	31	*A. baumannii* DSM30007	(Clifford et al. 2012)
E. coli	Fwd: GCATCGTGACCACCTTGA<br>Rev: CAGCGTGGTGGCAAAA	F(x)= -3.361x+35.797	59 bp	98.4 %	0.994	4	*E. coli* DSM1103	(Clifford et al. 2012)
**antibiotic resistance genes**								
*erm*B	Fwd: TGAATCGAGACTTGAGTGTGCAA<br>Rev: GGATTCTACAAGCGTACCTT	F(x)=-3.328x+35.901	71 bp	100 %	1.000	16	*S. hyointestinalis* DSM20770	(Alexander et al. 2015)
*IntI*1	Fwd: GCCTTGATGTTACCCGAGAG<br>Rev: GATCGGTCGAATGCGTGT	F(x)= -3.472x+34.720	196 bp	94.1 %	1.000	126	*E. coli* pNORM	(Rocha et al. 2018)
*tet*M	Fwd:GGTTTCTCTTGGATACTTAAATCAATC<br>Rev: CCAACCATAAATCCTTGTTCRC	F(x)=-3.424x+38.747	88 bp	95.9 %	0.998	4	*E. coli* DH5α	(Peak et al. 2007)
bla_{TEM}	Fwd: TTCCTGTTTTTGCTCACCCAG<br>Rev: CTCAAGGATCTTACCGCTGTTG	F(x)=-3.303x+38.559	112 bp	100.8 %	0.999	80	*E. coli* pNORM	(Rocha et al. 2018)
*sul*1	Fwd: CGCACCGGAAACATCGCTGCAC<br>Rev: TGAAGTTCCGCCGCAAGGCTCG	F(x) -3.387x+39.802	161 bp	97.6 %	0.999	80	*E. coli* pNORM	(Rocha et al. 2018)
$bla_{CTX\text{-}M15}$	Fwd: CGCTTTGCGATGTGCAG<br>Rev: ACCGCGATATCGTTGGT	F(x)= -3.504x+34.255	551 bp	92.9 %	1.000	93	*E. coli* pNORM	(Paterson et al. 2003, Rocha et al. 2018)
$bla_{CTX\text{-}M\text{-}32}$	Fwd: CGTCACGCTGTTGTTAGGAA<br>Rev: CGCTCATCAGCACGATAAAG	F(x)= -3.517x+37.800	155 bp	92.5 %	1.000	235	*E. coli* pNORM	(Rocha et al. 2018)
$bla_{OXA\text{-}48}$	Fwd: TGTTTTTGGTGGCATCGAT<br>Rev: GTAAMRATGCTTGGTTCGC	F(x)= -3.540x+36.913	177 bp	91.6 %	0.998	92	*K. pneumoniae* TGH Isolate 2	(Monteiro et al. 2012)

target	primer sequence	calibration curve	amplicon	efficiency	R^2	LOD	reference strain	literature
$bla_{\text{CMY-2}}$	Fwd: CGTTAATCGCACCATCACC<br>Rev: CGTCTTACTAACCGATCCTAGC	F(x)= -3.591x+34.026	172 bp	89.9 %	0.998	71	*K. pneumoniae* NRZ-01013	(Kurpiel and Hanson 2011)
*van*A	Fwd: TCTGCAATAGAGATAGCCGC<br>Rev: GGAGTAGCTATCCCAGCATT	F(x)= -3,541x+33,078	376 bp	91.6 %	1.000	43	*E. faecium* B7641	(Klein et al. 1998)
*mec*A	Fwd: CGCAACGTTCAATTTAATTTTGTTAA<br>Rev: TGGTCTTTCTGCATTCCTGGA	F(x)=-3,327x+34,887	91 bp	99.8 %	1.000	11	*S. aureus* A1	(Volkmann et al. 2004)
mcr-1	Fwd: GGGCCTGCGTATTTTAAGCG<br>Rev: CATAGGCATTGCTGTGCGTC	F(x)=-3,386x+35,349	183 bp	97.4 %	0.999	8	*E. coli* NRZ-14408	(Hembach et al. 2017)
$bla_{\text{NDM-1}}$	Fwd: TTGGCCTTGCTGTCCTTG<br>Rev: ACACCAGTGACAATATCACCG	F(x)= -3,293x+35,877	82 bp	101.2 %	0.999	66	*K. pneumoniae* ATCC BAA-2146	(Monteiro et al. 2012)

SI Table 5: wastewater treatment setup of investigated WWTPs

WWTP acronym	WWTP feed	primary treatment		secondary treatment			
C1	two-pipe drainage system	rake, sedimentation with skimmers		activated sludge treatment	P-elimination	secondary clarification	
C2	two-pipe drainage system	rake, sedimentation with skimmers		activated sludge treatment and enhanced biological P-removal	P-elimination	secondary clarification	
C3	two-pipe drainage system	rake, sedimentation with skimmers		activated sludge treatment	P-elimination	secondary clarification	
C4	two-pipe drainage system	rake, sedimentation with skimmers	primary clarification	activated sludge treatment	P-elimination	secondary clarification	
C5	two-pipe drainage system	rake, sedimentation with skimmers		activated sludge treatment	P-elimination	secondary clarification	
C6	two-pipe drainage system	rake, sedimentation with skimmers		activated sludge treatment	P-elimination	secondary clarification	
C7	two-pipe drainage system	rake, sedimentation with skimmers	primary clarification	activated sludge treatment	P-elimination	secondary clarification	

C8	two-pipe drainage system	rake, sedimentation with skimmers		activated sludge treatment	P-elimination	secondary clarification	
C9	two-pipe drainage system	rake, sedimentation with skimmers		activated sludge treatment	P-elimination	secondary clarification	
C10	two-pipe drainage system	rake, sedimentation with skimmers		activated sludge treatment	P-elimination	secondary clarification	
C11	two-pipe drainage system	rake, sedimentation with skimmers		activated sludge treatment	P-elimination	secondary clarification	
F1	two-pipe drainage system	rake, sedimentation with skimmers		activated sludge treatment	P-elimination	secondary clarification	
F2	two-pipe drainage system	rake, sedimentation with skimmers		activated sludge treatment	P-elimination	secondary clarification	
F3	two-pipe drainage system	rake, sedimentation with skimmers		activated sludge treatment	P-elimination	secondary clarification	
F4	two-pipe drainage system	rake, sedimentation with skimmers		activated sludge treatment and enhanced biological P-removal	P-elimination	secondary clarification	
H1	two-pipe drainage system	rake, sedimentation with skimmers		activated sludge treatment	P-elimination	secondary clarification	
H2	mixed sewer system	rake, sedimentation with skimmers		activated sludge treatment	P-elimination	secondary clarification	
H3	mixed sewer system	rake, sedimentation with skimmers	primary clarification	activated sludge treatment	P-elimination	secondary clarification	
H4	two-pipe drainage system	rake, sedimentation with skimmers		activated sludge treatment	P-elimination	secondary clarification	
H5	two-pipe drainage system	rake, sedimentation with skimmers	primary clarification	activated sludge treatment and enhanced biological P-removal	P-elimination	secondary clarification	
H6	two-pipe drainage system	rake, sedimentation with skimmers		activated sludge treatment	P-elimination	secondary clarification	

H7	two-pipe drainage system	rake, sedimentation with skimmers		activated sludge treatment	P-elimination	secondary clarification	
H8	mixed sewer system	rake, sedimentation with skimmers		activated sludge treatment	P-elimination	secondary clarification	cloth filter
